# Identification of neutralising pembrolizumab anti-drug antibodies in patients with melanoma

**DOI:** 10.1038/s41598-021-98700-7

**Published:** 2021-09-28

**Authors:** S. C. Sasson, L. E. Wilkins, R. A. Watson, C. Jolly, O. Brain, P. Klenerman, A. Olsson-Brown, B. P. Fairfax

**Affiliations:** 1 Nuffield Department of Medicine, The University of Oxford, Oxford, UK; 2 The University of Oxford Medical School, Oxford, UK; 3 MRC Weatherall Institute of Molecular Medicine, The University of Oxford, Oxford, UK; 4 The Clatterbridge Cancer Centre, Liverpool, UK

**Keywords:** Immunotherapy, Immunology, Cancer, Cancer therapy

## Abstract

Development of anti-drug antibodies (ADAs) can interfere with therapeutic monoclonal antibodies and may lead to drug neutralisation and clinical disease progression. Measurement of circulating drug levels and development of ADAs in the setting of anti-programmed cell death-1 agent pembrolizumab has not been well-studied. Enzyme-linked immunosorbent assays were used to measure pembrolizumab drug level and ADAs in 41 patients with melanoma at baseline, Time-point 1 (3 weeks) and Time-point 2 (21 weeks). Assay results were related to patient demographics and clinical outcome data at 6 months. The median pembrolizumab drug level at 3 weeks was 237 ng/μL and did not correlate with age, sex or body surface area.17/41 patients had an ADA detected at any timepoint, with the highest prevalence at Timepoint 1 (median concentration = 17 ng/μL). The presence of an ADA did not correlate with clinical progression at 6 months. 3/41 (7%) of patients displayed a falling pembrolizumab drug level and rising ADA titre between Timepoint 1 and 2 suggestive of a neutralising ADA. Pembrolizumab drug levels and ADAs can be readily measured. The rates of total and treatment-emergent ADAs may be higher in “real-word” settings than those previously reported. Larger studies are needed to determine effect of neutralising ADAs on long-term clinical outcome.

## Introduction

The use of therapeutic monoclonal antibodies has grown over recent years, particularly for haematological and solid organ malignancies and chronic autoimmune and inflammatory conditions. The efficacy of these treatments can be affected by the development of a host immune response and the production of anti-drug anti-bodies (ADAs) that can reduce circulating drug levels and negatively impact on clinical outcomes (reviewed in^1,2^).

Development of ADAs has been best characterised in the setting of the anti-tumour necrosis factor (TNF)-α drugs adalimumab, infliximab and golimumab used to treat autoimmune arthritides and enteropathies. The frequency of ADA formation varies between agents, with a higher rate of development with infliximab (rage 0–83% of patients) compared with adalimumab (0–54%) and golimumab (0–19%)^1^. ADA development has also been reproducibly reported in the setting of abatacept, certolizumab, etanercept, rituximab, secukinumab, tocilizumab and ustekinumab. The antigenic site is largely related to the antibody binding fragment, however ADAs targeting the antibody hinge regions of abatacept and etanercept have also been described^1^.

A systematic review of patients treated with TNF-α blockade found that overall the presence of ADAs was associated with poor outcome and was more likely to develop in females^3^. Co-administration of methotrexate may decreased the rate of ADA formation^3^. Such findings have translated into clinical management of inflammatory bowel disease (IBD) prescribed TNF-α blockade. Treated patients with symptoms of clinical flare undergo therapeutic drug monitoring. If sub-therapeutic levels of drug are found, serum should be tested for anti-TNF-α ADAs. The combination of low drug levels and a positive ADA test is interpreted as indication to switch to a different anti-TNF-α antibody or class of drug^4^.

Immune checkpoint inhibitors that target Programmed Cell Death-1 (PD-1; pembrolizumab and nivolumab) were initially licensed for use in metastatic melanoma, and are now approved for use in renal cell carcinoma and small cell lung cancer, with further trials underway. Anti-PD-1 therapy can be given in combination with anti-CTLA-4 or as monotherapy. Anti-PD-1 is generally well tolerated and has a lower rate of immune-related adverse events (irAE), leading to its increased use particularly in more elderly or frail patients. Unlike anti-CTLA-4 therapy, which is given for a short period of time, anti-PD-1 therapy can be given for extended periods of time and even out to 2 years. Despite the widespread uptake of anti-PD-1 therapy there are limited studies on therapeutic drug monitoring and/or development of anti-drug antibodies, particularly in real-world clinical settings. Additionally, PD-1 expression is high on follicular helper T cells^5^, which augment B cell production of antibodies in the lymph-node and it is unclear if anti-PD-1 therapy could promote high amounts of ADAs. A proportion of oncology patients do not respond to pembrolizumab and the role of pembrolizumab ADAs in this unresponsiveness remains unclear.

Pembrolizumab ADAs have been reported to occur at a rate 0.7–2.5%^6,7^ in the setting of monotherapy, and can be associated with infusion-related hypersensitivity reactions^8^. van Vugt et al. studied 3655 patients receiving pembrolizumab monotherapy for a range of malignancies using various regimens^9^. In advanced melanoma treated with 200 mg of pembrolizumab every 3 weeks, the rate of total and treatment-emergent ADAs were 5.6% and 1.9%, respectively^9^. There is limited large-scale data on the development of pembrolizumab neutralising ADAs. In the aforementioned study the rate of neutralising treatment emergent ADA in the melanoma sub-group was 0%^9^.

Recently two research assays were released for (1) therapeutic drug monitoring of pembrolizumab and (2) pembrolizumab ADAs. In this study we sought to evaluate the usefulness of these assays in measuring drug levels and ADAs in patients being treated with pembrolizumab monotherapy for melanoma in “real-world” settings at two UK centres. We sought to understand if either drug levels or ADAs were related to clinical outcomes at 6 months of treatment.

## Methods

### Patients

41 patients diagnosed with melanoma and treated with pembrolizumab (200 mg every 3 weeks) monotherapy as first-line agent were recruited from Oxford and Liverpool, UK. All patients provided written informed consent for sample collection and research participation. This study was approved by the South Central-Oxford C Research Ethics Committee (Reference 19/SC/0173) and the North West Greater Manchester West Research Ethics Committee (Reference 12/NW/0525), both part of the National Health Service, United Kingdom. Samples were taken at baseline i.e. before the administration of pembrolizumab, Time-point 1 (just before the second dose) and Timepoint 2 (last available sample). The median time for Time-point 2 was just prior to Cycle 7 (21 weeks). Patients recruited in Oxford had plasma analysed while those in Liverpool had serum analysed as these were the samples stipulated in the established research protocols. All research involving human participants was performed in accordance with the Declaration of Helsinki.

### Pembrolizumab drug level assay

**Figure 1:**
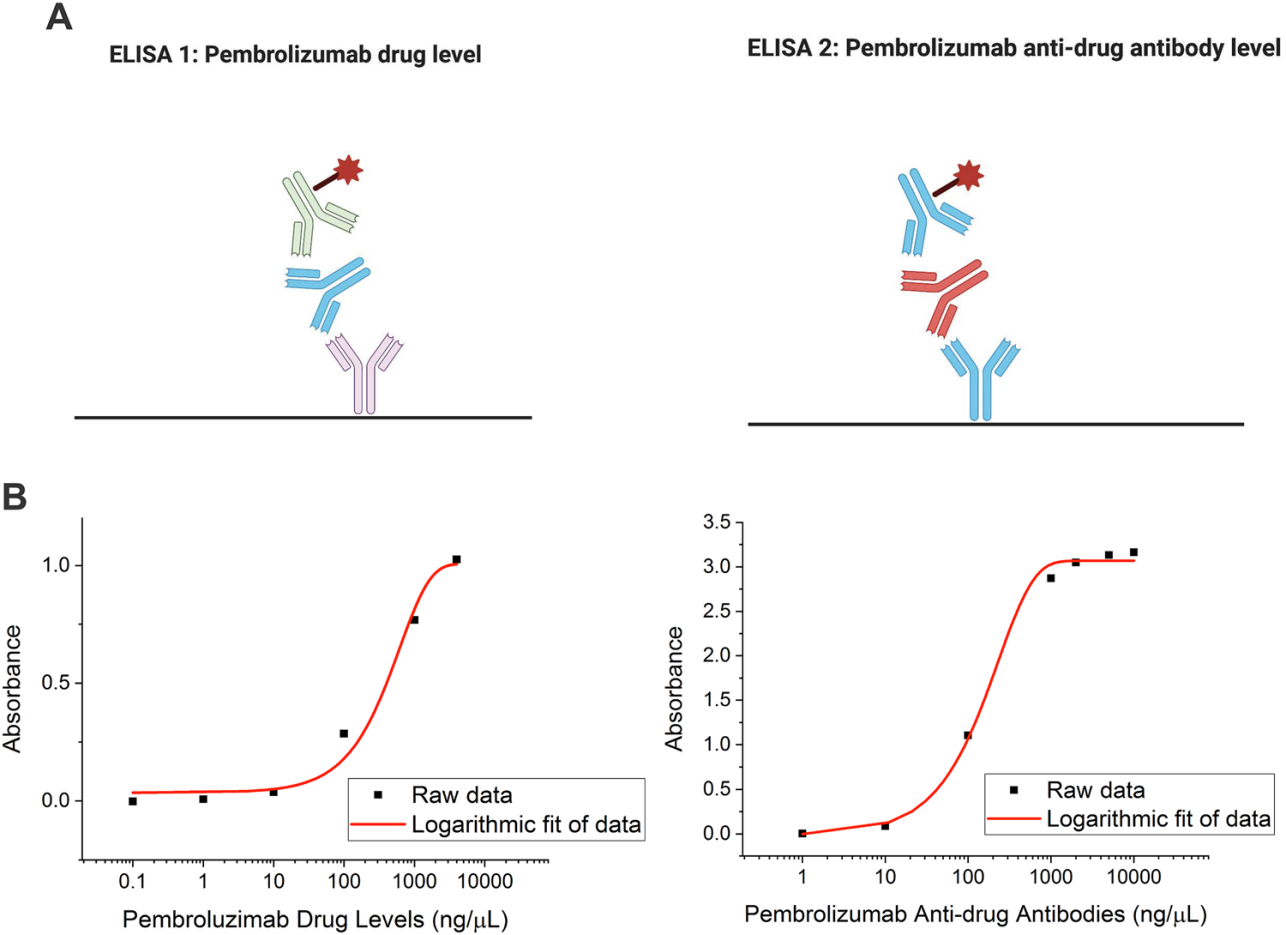
**Pembrolizumab drug level and anti-drug anti-bodies: assay overview and standard curves**. (**A**) Schematic of (left) pembrolizumab drug level assay. Sandwich enzyme-linked immune-sorbent assay (ELISA) with capture antibody (purple) that binds patient serum-derived pembrolizumab (blue). Detection antibody (green) is conjugated to horseradish peroxidase (HRP; red star) facilitating colormetric quantitation. Pembrolizumab anti-drug anti-body (ADA) assay (right) was achieved by sandwich ELISA using commercial pembrolizumab capture antibody (blue) to detect patient serum-derived ADA (red). Detection antibody was commercial pembrolizumab (blue) conjugated to HRP (red star). (**B**) Performance of commercial standard curved for pembrolizumab drug level (left) and ADA (right) are shown.

Circulating pembrolizumab levels were measured according to manufacturers instructions (Bio-Rad, Hercules, CA, USA). Briefly, anti-pembrolizumab capture antibody was diluted to 1 μg/mL in phosphate-buffered saline (PBS) and coated on Nunc Maxisorp™ 96-well plates (Thermo Fisher Scientific, Waltham, MA, USA) overnight at 4 °C. Following washing with PBS/0.05% Tween-20(Sigma, St Louis, MO, USA) plates were blocked using SuperBlock™ reagent(Thermo Fisher Scientific). 100 μL of pembrolizumab (Merk&Co, Kenilworth, NJ, USA) standard (0–4000 ng/μL) or plasma/serum was incubated for 1 h at RT. Following washing, horseradish peroxidase (HRP)-conjugated 2 μg/mL anti-pembrolizumab detection antibody (Bio-Rad) was added for 1 h at RT. Following washing, assay was developed using 3,3′,5,5′ tetramethylbenzidine (TMB) substrate (Biolegend, San Diego, CA, USA) for 30 min prior to stopping. Colouremetric change was measured at 450 nm using a FLUOStar microplate reader (BMG Labtech, Ortenburg, Germany). Pembrolizumab drug level assay overview and a representative standard curve are shown in Fig. 1.

### Anti-drug antibody assay

Pembrolizumab ADA levels were measured according to manufacturers instructions (Bio-Rad). Briefly, pembrolizumab (Meck & Co) was utilised as a capture antibody was diluted to 1 μg/mL in phosphate-buffered saline (PBS) and coated on Nunc Maxisorp™ 96-well plates (Thermo Fisher Scientific) overnight at 4 °C. Following washing with PBS/0.05% Tween-20 (Sigma) plates were blocked using SuperBlock™ reagent(Thermo Fisher Scientific). 100 μL of assay standard (Bio-Rad; 0–10,000 ng/μL) or plasma/serum was incubated for 1 h at RT.

For this assay HRP was conjugated to pembrolizumab for use as a detection antibody, using the LYNX Rapid conjugation kit (Bio-Rad). Following washing, HRP-pembrolizumab was added for 1 h at RT. Following washing, assay was developed using TMB substrate (Biolegend) for 30 min prior to stopping. Colourmetric change was measured at 450 nm using a FLUOStar microplate reader (BMG Labtech). Pembrolizumab ADA assay overview and a representative standard curve are shown in Fig. 1.

### Statistics

Standard curve and equation derivation were determined from logarithmic curve fitting in OriginPro 2018b Software (OriginLab, Northampton, MA, USA). Differences across three timepoints were determined using 1-way ANOVA. Differences between two categories were determined using the non-parametric Mann–Whitney test. Relationship between two continuous variables were determined using the non-parametric Spearman’s correlation. *P* values < 0.05 were considered significant. All tests were performed using GraphPad Prism v8.0 (San Diego, CA, USA).

## Results

### Patient characteristics

Patient characteristics are shown in Table 1. The cohort were predominantly male (56%) with a median age of 73 years. 85% of patients had Stage IV disease at baseline and 17% had progressive disease at 6 months.

**Table 1:** Patient characteristics.

Total number of patients	41
Male (%)	23 (56%)
Age (years)	73 (69–81)
Weight (kg)	87 (74–94)
Body surface area (m^2^)	2.03(1.83–2.11)
**Stage (AJCCv8)**	
IV	35 (85%)
III	6 (15%)
Progression at 6 months	7 (17%)

Median values shown (interquartile range).

### Pembrolizumab drug levels

**Figure 2:**
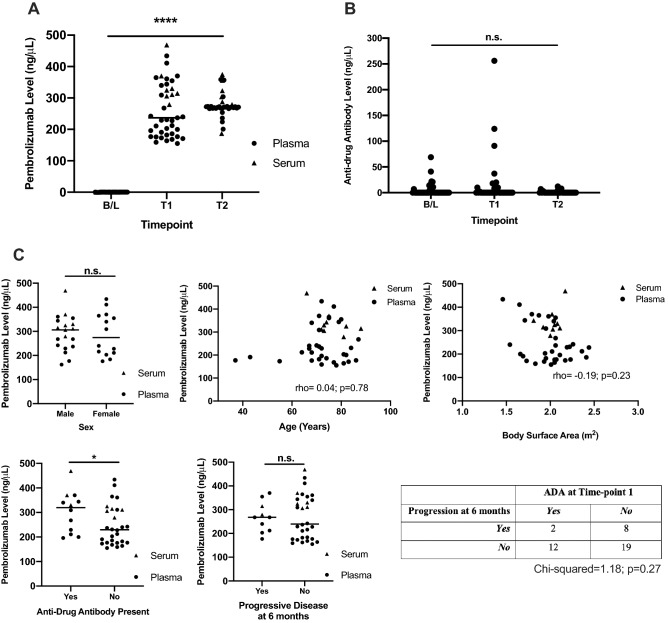
**Pembrolizumab drug and anti-drug antibody (ADA) levels in a clinical cohort**. Data generated from patients with melanoma treated with pembrolizumab (n=41). (**A**) Pembrolizumab drug level at baseline (B/L), just prior to Cycle 2 (T1) and last available timepoint (T2; Median Cycle 7 or 21 weeks IQR Cycle 4-13). (**B**) pembrolizumab ADA were detectable at B/L, T1 and T2. (**C**) Clinical correlates to pembrolizumab drug level just prior to Cycle 2 (T1). Pembrolizumab drug level did not significantly correlate with sex, age, body surface area or progressive disease at 6 months. Patients with an ADA at T1 had higher pembrolizumab drug levels (*p<0.05).

Circulating pembrolizumab drug levels are shown in Fig. 2A. There were no detectable drug levels at baseline i.e. prior to treatment. The median levels of pembrolizumab at Time-point 1 and Timepoint 2 were 237 ng/μL and 271 ng/μL respectively. Patients with stored serum had higher pembrolizumab drug levels than those with stored plasma (median serum 320 ng/μL inter-quartile range 307–360 ng/μL; median plasma 212 ng/μL inter-quartile range 177–325 ng/μL; *p* = 0.01). However, each individual patient had consecutive samples of only the same material i.e. only serum *or* plasma. There was no significant difference in circulating pembrolizumab based on sex, age or body surface area (Fig. 2Ci–iii). Patients with progressive disease at 6 months did not have significantly different drug levels compared with those that did not (Fig. 2Cv).

### Anti-drug anti-bodies and their correlates

Pembrolizumab ADAs were detectable at all three timepoints including at baseline i.e. prior to therapy (Fig. 2B). 17/41 patients had an ADA detectable at any timepoint. ADAs were detectable at baseline in 9/41 (median = 16 ng/μL), Timepoint 1 in 12/41 (median = 17 ng/μL) and Timepoint 2 in 5/41 (median = 7 ng/μL). At Timepoint 1 the presence of an ADA was associated with higher circulating drug levels of pembrolizumab (330 ng/μL vs 228 ng/μL *p* < 0.05; Fig. 2Civ). The rate of disease progression was no higher in patients with an ADA at time-point 1, compared to those without (Fig. 2C).

### Three patients with results consistent with a neutralising anti-drug antibody

**Figure 3:**
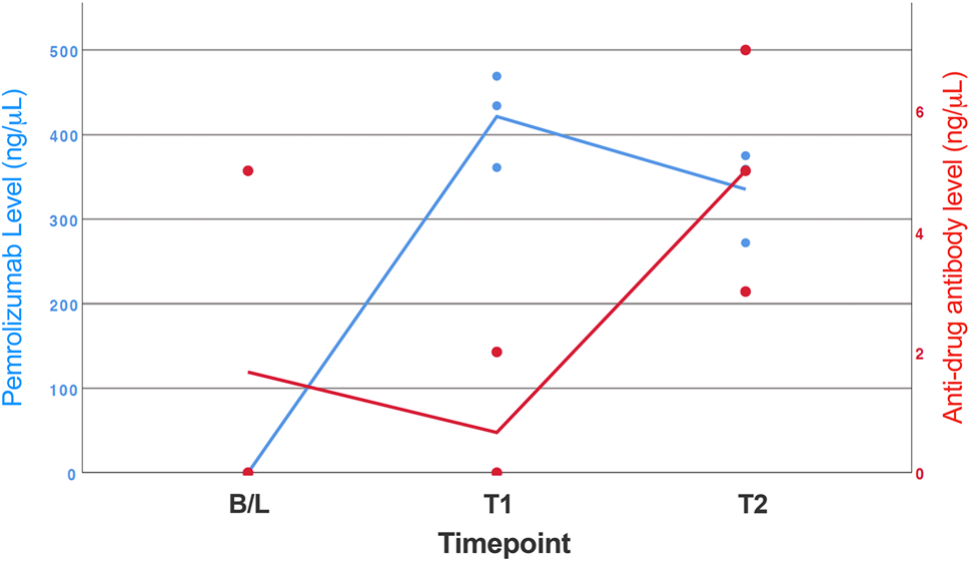
**Pembrolizumab drug and anti-drug antibody (ADA) levels in 3 patients with results suggestive of a neutralising ADA**. 3/41 patients (7%) with results suggestive of a neutralising ADA are shown. All 3 patients had falling pembrolizumab drug levels and rising ADA between Cycle 2 (T1) and last available time-point (T2; Median Cycle 7 or 21 weeks IQR Cycle 4-13).

3/41 patients (7%) had falling pembrolizumab drug levels and rising ADA levels in between Timepoint 1 and 2, findings compatible with the development of a neutralising ADA. In these three patients the median drug level fell from 434 to 359 ng/μL and the ADA rose from a median of 0 to 3 ng/μL (Fig. 3). The clinical data from these patients is shown in Table 2.

**Table 2:** Characteristics of patients with falling pembrolizumab drug level and rising anti-drug antibody level.

Patient	Sex	Age (years)	Stage	Pembrolizumab level B/L (ng/μL)	Pembrolizumab level T1 (ng/μL)	Pembrolizumab level T2 (ng/μL)	ADA level B/L (ng/μL)	ADA level T1 (ng/μL)	ADA level T2 (ng/μL)	Progression at 6 months
1	M	66	4	0	469	375	0	2	5	Partial response
2	M	75	4	0	361	272	5	0	7	No Progression
3	F	72	4	0	434	359	0	0	3	No Progression

## Discussion

The expanding development and clinical use of therapeutic monoclonal antibodies has transformed the management of patients with specific haematological and solid organ malignancies as well as those with autoimmune and inflammatory conditions. Optimal use of these therapies will rely on an understanding of the development of ADAs which have the potential to neutralise the therapeutic mAb and negatively impact on clinical outcomes. Here we investigated circulating drug levels and ADAs in patients being treated with pembrolizumab, using two recently released research assays. Pembrolizumab is being used for an expanding number of indications and often for periods of up to two years, and therefore we postulated it would be important to recognise the development of ADAs.

The drug level assay performed well, with no detection of pembrolizumab at baseline (i.e. prior to treatment), indicating little to no cross-reactivity. Pembrolizumab levels were not correlated to age, sex, body surface area or progression at 6 months.

A striking finding was that pembrolizumab ADAs were measurable at all three study timepoints with an overall frequency of 41%, far higher than previously published reports of up to 2.5–5/6%^6,9^. While the higher frequency obtained in this study may be a function of smaller sample size, it also raises the possibility that some subgroups of patients more likely to develop ADAs may have been excluded from the initial licensing studies from which the earlier studies draw. The finding that pembrolizumab ADAs can be present at baseline, prior to drug administration, indicates that antibodies that bind pembrolizumab can pre-exist in patients prior to treatment, and this has been demonstrated in other studies^9^. The frequency and concentration of pembrolizumab ADAs was highest at Timepoint 1, just prior to Cycle 2 of treatment. At Timepoint 1, the present of ADA was associated with higher circulating drug levels, raising the possibility that ADAs have the potential to increase drug half-life, and this will require further confirmatory studies. The presence of an ADA just prior to Cycle 2 of treatment was not associated with increased rate of progression at 6 months. This finding is in line with the largest published study to-date of pembrolizumab ADAs which followed patients out to 500 days^9^.

Our results demonstrate that a minority of patients (7%) develop falling drug levels and rising ADA levels, consistent with a neutralising ADA. This is an important finding as rates of neutralising ADAs have not been widely reported. The concentration of the neutralising ADAs can be relatively low. Again, the presence of a neutralising ADA did not predict clinical progression at 6 months, in line with previous reports^9^.

In conclusion, we have investigated two recently released research assays for monitoring of pembrolizumab drug levels and ADAs. This data is a useful contribution to the field however our approach has several limitations. Firstly our cohort size is relatively small compared to the number of patients being treated with pembrolizumab. Commensurably, only a small number of our cohort (17%) had clinical progression at 6 months, and we may have been underpowered to detect significant correlates to this outcome. Additionally, around a quarter of patients had serum bio-banked while the remainder has plasma, although there was consistency in the sample type analysed for individual patients, and therefore differences here do not explain longitudinal trends. Further studies involving larger cohorts of patients over a longer period of follow-up may be necessary to determine the long-term effects of pembrolizumab ADAs on clinical outcomes.
